# Investigating molecular descriptors in cell-penetrating peptides prediction with deep learning: Employing N, O, and hydrophobicity according to the Eisenberg scale

**DOI:** 10.1371/journal.pone.0305253

**Published:** 2024-06-13

**Authors:** Juliana Auzier Seixas Feio, Ewerton Cristhian Lima de Oliveira, Claudomiro de Souza de Sales, Kauê Santana da Costa, Anderson Henrique Lima e Lima

**Affiliations:** 1 Laboratório de Inteligência Computacional e Pesquisa Operacional, Campus Belém, Instituto de Tecnologia, Universidade Federal do Pará, Pará, Brazil; 2 Laboratório de Simulação Computacional, Campus Marechal Rondom, Instituto de Biodiversidade, Universidade Federal do Oeste do Pará, Santarém, Pará, Brazil; 3 Laboratório de Planejamento e Desenvolvimento de Fármacos, Instituto de Ciências Exatas e Naturais, Universidade Federal do Pará, Belém, Pará, Brazil; 4 Instituto Tecnológico Vale, Belém, Pará, Brazil; Jeonbuk Natiomal University, KOREA, REPUBLIC OF

## Abstract

Cell-penetrating peptides comprise a group of molecules that can naturally cross the lipid bilayer membrane that protects cells, sharing physicochemical and structural properties, and having several pharmaceutical applications, particularly in drug delivery. Investigations of molecular descriptors have provided not only an improvement in the performance of classifiers but also less computational complexity and an enhanced understanding of membrane permeability. Furthermore, the employment of new technologies, such as the construction of deep learning models using overfitting treatment, promotes advantages in tackling this problem. In this study, the descriptors nitrogen, oxygen, and hydrophobicity on the Eisenberg scale were investigated, using the proposed ConvBoost-CPP composed of an improved convolutional neural network with overfitting treatment and an XGBoost model with adjusted hyperparameters. The results revealed favorable to the use of ConvBoost-CPP, having as input nitrogen, oxygen, and hydrophobicity together with ten other descriptors previously investigated in this research line, showing an increase in accuracy from 88% to 91.2% in cross-validation and 82.6% to 91.3% in independent test.

## Introduction

Therapeutic molecules face challenges in entering the intracellular environment due to selective control of molecule permeability through cell membranes, affecting passive diffusion and active transport [1]. Predicting therapeutic peptides is crucial for research in peptide-based therapies, bringing recent advances in these molecule synthesis improving their potency, specificity, and permeability, and thereby increasing their use in the pharmaceutical industry [2].

Peptides are biologically active and structurally heterogeneous molecules showing different physicochemical and structural properties [3, 4], playing essential roles in regulating cellular functions, besides showing interesting biological activities [5]. Cell-penetrating peptides (CPPs) can naturally cross the lipid bilayer membrane that protects cells, containing amino acid sequences ranging from 5 to 42, being water-soluble, partially hydrophobic, often cationic or amphiphilic, and rich in arginine and lysine residues [6].

Due to their high structural complexity and chemical versatility, several studies have focused on predicting their mechanism and efficiency of transport and penetration through cell membranes, also addressing dimensionality reduction techniques to correlate molecular descriptors or select high-impact features in predicting cell-penetrating peptides [7, 8]. Many functional peptides identified provide a wealth of detailed data that allows researchers to predict functional peptides with machine learning algorithms (ML), reducing the time of labor in biological experiments and giving cost-effective predictions [9].

Dobchev et al. (2010) [10] and Sanders et al. (2011) [11] were the first to use ML to predict CPPs by exploring different techniques and several molecular descriptors. Since then, researchers have been studying this topic by developing CPP classifiers. However, more recent studies—from 2020 to the present—have focused on developing tools for CPP predictions and identifying the most relevant descriptors to classify this group of peptides using feature compositions (FCs).

In this context, the studies of Xiangzheng et al. (2020) [12] aimed to predict CPPs by proposing the StackCPPred framework with three feature extraction methods based on the paired residue energy content: composition-RECM (residue energy content matrix), pseRECM (pseudo residue energy content matrix) and RECM-DWT (residue energy content matrix—discrete wavelet transform). The authors used CPP924 and CPPsite3 datasets [13, 14]. As a result, 60 most important resources belonging to FC PseRECM were listed—FC most relevant in the correct classification of CPPs in this work. On the CPP924 dataset, the StackCPPred framework achieved 94.5% accuracy using all FCs. However, evaluating the compositions individually, the accuracies obtained were between 66.94% and 77.79% [12], even after pairwise combinations of some FCs to generate new sets of resources.

Arif et al. (2020) [15] developed a method to discriminate CPPs and non-CPPs (non-cell membrane penetrating peptides) called TargetCPP using Gradient Boosting Decision Tree Classifier. The authors generated FCs according to 1) composite protein sequence representation, 2) composition distribution and transition, 3) split amino acid composition, and 4) feature information theory for feature selection. Despite obtaining an improvement in all indicators due to a selection of features, with accuracy improving from 90.48% to 93.54% in cross validation, these results were obtained only with the union of all 80 features, while the FCs achieved between 80.4% and 87% accuracy individually [15].

Oliveira et al. (2021) [16] proposed an ML-based framework called BChemRF-CPPred that uses an ANN, a Support Vector Machine (SVM) and a Gaussian process classifier (GPC) to differentiate CPPs from non-CPPs. In this work, sequence and structure-based descriptors were covered, in addition to PDB and FASTA formats. The dataset containing an optimized selection of 43 sequence- and structure-based descriptors was identified by the authors as the best for predicting CPPs, as the developed framework achieved, respectively, cross-validation and independent testing accuracies of 87.66% and 90.60% for the PDB input format, and 86.9% and 86.5% for the FASTA format [16]. However, the less complex set (with 12 descriptors) obtained the worst results, demonstrating that the proposed classifier needs the largest number of descriptors to perform well.

Several ML methods have proven to be an efficient approach for selecting, filtering, and predicting biomolecule properties, providing accurate predictions, improving drug development decisions, and clarifying the chemical properties of these compounds [17–20].

However, artificial intelligence approaches, such as Deep Learning (DL) have been widely used to predict biological activities due to their ability to detect complex chemical patterns that traditional statistical methods struggle to capture [19, 21]. Among DL methods, deep neural networks are currently the most advanced approaches, and convolutional neural networks (CNNs), in particular, have shown remarkable performance in various challenging computational tasks [21].

CNNs are a class of DL architectures that have shown promising results and have gained prominence not only in image analysis but also in tabular data. One of the main advantages of CNN is its high efficiency, meaning less training time and samples are needed to achieve a good level of performance. This class of ANN also requires fewer neurons due to the convolutional architecture being able to process data for small sub-areas, allowing the network to be deeper and have fewer parameters, whose receptive fields share coefficients and bias, reducing memory consumption [22].

By introducing CNNs into peptide classification, Zhang et al. (2022) [9] developed a model to differentiate eight different categories of therapeutic peptides, including CPPs. In this study, the results of the developed neural network were compared with a deep neural network, a simple CNN, Long Short Term Memory (LSTM), SVM, Random Forest (RF), and XBoost. The feature extraction methods 1) binary coding, 2) enhanced group amino acid composition, 3) group weight-based coding, 4) BLOSUM62 matrix and 5) K-Nearest Neighbors (KNN) were used to extract data from peptides sequences, achieving an accuracy of 96.84% for CPPs in ten-fold cross-validation with a combination of the five FCs. However, the FCs were unable to achieve optimal results when evaluated individually, achieving accuracy between 74% and 88%, as well the extraction did not make it possible to discriminate the molecular descriptors with greater relevance in the classification [9].

Park et al. (2023) [23] developed a method for predicting CPPs based on DL called AiCPP with the aim of avoiding false-positive results, incorporating the LSTM algorithm. This work contributes to research into predictions of new CPPs with the discovery that short peptide sequences derived from amyloid precursor proteins are more efficient in permeating the cell membrane, despite not achieving the objective of reducing false-positive results in relation to other algorithms, with 88.6% accuracy against more than 90% of this metric achieved by other algorithms [23].

Therefore, depending solely on deep or machine learning techniques does not guarantee superior prediction of CPPs. Molecular descriptors derived from peptides may more accurately depict the chemical information encoded by the molecular mechanism of cell membrane penetration. Consequently, these descriptors can aid in enhancing the mathematical and logical procedures used for predicting the pharmacokinetic and biological properties of these structures [12, 24, 25].

The result of a statistical study by Dichiara et al. (2020) [26] on 15 molecular descriptors of blood-brain barrier penetrating peptides showed that 9 of these descriptors have a highly significant *χ*^2^ distribution: polar surface area, nitrogen and oxygen count, logP, nitrogen count, logD, oxygen count, ionization state, hydrogen bond acceptors, and hydrogen bond donors [26].

Another molecular descriptor addressed in research on peptides is hydrophobicity. When the atomic coordinates of a protein segment are known, the hydrophobic moment (called the structural hydrophobic moment) can be calculated as follows [27]:

$$Ho=\sum_{n=0}^{\infty }H_{n}S_{n}$$ (1)

where *H*_*n*_ represents the hydrophobicity of the nth residue in the protein segment, while *S*_*n*_ is the unit vector pointing from the *α* carbon atom to the center of the residue side chain. Thus, the hydrophobic moment is a measure of the sum of the directions of the amino acid side chains, with each direction weighted by the hydrophobicity of the corresponding amino acid [27].

Porto et al. (2022) [28] hypothesized that the hydrophobic moment could be used as a classifier for Antimicrobial peptides (AMPs) and selected the Eisenberg scale to calculate the hydrophobic moment of peptides. As result, they concluded that hydrophobic moment discriminates AMPs better, being comparable to DL approaches and reaching accuracy greater than 80% and sensitivity greater than 90% [28].

These works motivate the investigation of these descriptors by applying them to CPPs for accuracy improvement by observing such molecular properties. Specifically, this work aims to investigate combinations of some sequence- and structure-based molecular descriptors by adding nitrogen counts (N), oxygen counts (O), and hydrophobicity on the Eisenberg scale (Ho), performing the CPP classification through an ensemble model containing DL and a ML algorithm. Classification results are analyzed in terms of FCs with each other and comparisons with other previously published FCs for CPP classification.

## Materials and methods

### Dataset

Unlike the previously mentioned works that use mostly primary structure files (FASTA), the focus of this work is on the use of tertiary structure files containing peptide information called PDB (Protein Data Bank)—a standard format for files containing atomic coordinates [29]. With this peptide data structure, it is possible to extract more information about the structure of molecules than FASTA format [29].

Although this format is considered difficult to obtain and use, there are currently some computational tools that have been developed to predict the tridimensional structure of peptides, such as Pep-fold server [30] and PEPstrMOD [31]. In addition, some computational tools calculate molecular properties through PDB files, containing non-natural or modified residues, such as BChemRF-CPPred webserver [16].

The peptide structures were obtained from two curated and validated databases. The structures of CPPs were obtained from CPPsite2.0 [32] and C2Pred [33]. Additionally, other structures were obtained from previously published studies and pharmaceutical catalogs [11, 34, 35]. In the preprocessing stage, peptides were filtered for length, with only those containing between 5 and 30 amino acid residues being kept, since CPPs are a class of small peptides 5–30 amino acids in length [36]. Also, duplicate structures were removed using the Pandas library [37] for the Python language [38].

The XGBoost and CNN models were trained and validated using ten-fold cross-validation on a dataset comprising 600 peptide structures (300 CPPs and 300 non-CPPs). For independent testing, an additional 150 structures (75 CPPs and 75 non-CPPs) were utilized. Both training and testing sets consist of natural and modified peptides.

### Feature compositions

In this study, structure- and sequence-based descriptors are investigated as two classes of molecular descriptors. The 12 structure-based descriptors include structural and physicochemical properties related to the permeation of molecules in biological membranes, namely: molecular weight (MW), topological polar surface area (tPSA), number of rotatable bonds (NRB), fraction of *sp*^3^-hybridized carbon atoms (*Fsp*^3^), water/1-octanol partition coefficient (cLogP), number of hydrogen bond donors (HBD), number of hydrogen bond acceptors (HBA), number of aromatic rings (NAR), and net charge (NetC), according to [39–41]. In addition to these descriptors, some properties related to charged polar groups were also used in the FCs: primary amine groups (NPA), number of guanidine groups (NG), and number of negatively charged amino acid groups (NNCAA) [7, 14, 42].

The other class of molecular properties comprises the sequence-based descriptors, which represent the information calculated from the primary structure of the peptide, consisting of amino acid composition (AAC), formed by two amino acid compositions related to the fractions of arginine and lysine [6, 43]; 22 pseudo amino acid composition (PseAAC), formed by a combination of discrete sequence correlation factors and twenty components of the conventional amino acid composition [44]; and 40 dipeptide compositions (DPC), to evaluate the presence of motifs in CPP sequences that are relevant for their cell uptake mechanism [7, 14, 42, 45, 46]. Firstly, the FCs are generated as follows:

1. “FC-SEQ” (64 ***seq*** *uence-based* descriptors): containing only sequence-based descriptors (AAC, PseAAC, and DPC);
2. “FC-STR” (12 ***str*** *ucture-based* descriptors): containing only structure-based descriptors (MW, tPSA, *Fsp*^3^, cLogP, HBA, HBD, NAR, NRB, NetC, NPA, NG, and NNCAA);
3. “FC-SEQ-STR” (76 ***seq*** *uence-based and* ***str*** *ucture-based* descriptors): with the sequence- and structure-based descriptors; and
4. “FC-Kendall” (43 structure- and sequence-based descriptors): a selection of structure- and sequence-based descriptors according to Kendall’s correlation analysis, which includes all AAC and PseAAC, the top 10 correlated DPCs (Glycine-Leucine, Glycine-Phenylalanine, Leucine-Glycine, Glycine-Alanine, Valine-Cysteine, Arginine-Lysine, Arginine-Glutamine, Lysine-Arginine, Lysine-Lysine, Arginine-Arginine), and the structure-based descriptors excepting tPSA, NRB, and HBD.

Calculations of those molecular descriptors can be extracted from the web server BChemRF-CPPred available in comptools.linc.ufpa.br/BChemRF-CPPred/ [16], or they can be calculated with RDKit [47], Biopython [48] and PyBioMed [49] libraries in Python programming language [38]. Counts of O and N were calculated RDKit library [47], and Ho of the peptides was calculated on the Eisenberg scale using the PepFun framework [50]. Then, the new quantified descriptors were added to the FCs that contain structure descriptors, i. e. all FCs except FC-SEQ.

Afterward, a Kendall’s correlation analysis was made to evaluate the degree of similarity between the new descriptors and the old ones, nevertheless, it was also applied to FC-SEQ to treat other correlated descriptors. The outcomes were: 1) the descriptor HBD was removed from FC-STR and FC-SEQ-SRT, 2) the descriptor F[ARG] was removed from FC-Kendall and FC-SEQ, 3) the descriptor MW was removed from FC-STR, FC-SEQ-STR and FC-Kendall, and 4) N, O, and Ho were kept in the FCs where they were added after this analysis. Thus, the proposed FCs were generated containing 63, 13, 77, and 44 descriptors, respectively.

### The proposed ConvBoost-CPP

In this work, XGBoost and CNN were the algorithms selected to build a voting classifier, which was called ConvBoost-CPP. For the XGBoost algorithm, a Bayesian optimization over hyperparameters was made using the BayesSearchCV method from Scikit optimizer module [51]. BayesSearchCV implements a tuning and scoring method where searching over the hyperparameter settings of the used estimator is optimized by cross-validation [52]. The tuned hyperparameters were selected according to the problem—as it is a binary classification problem with balanced classes—and are detailed below.

#### XGBoost hyperparameter tuning

The eXtreme Gradient Boosting package—XGBoost—is widely used in data science research implementing the Gradient Boosting framework [53]. The XGBoost is a boosting technique that sequentially creates decision trees, each tree improving upon the mistakes of the previous one.

The hyperparameter *n_estimators* defines the number of trees to be built and *max_depth* determines how deep each tree can grow during a given round of boosting [53]. Such trees are constructed using a tree construction method that is defined by the hyperparameter *tree_method* whereas *min_child_weight* is the minimum weight required to create a new node in the tree [53]. Colsample_bytree is the subsample ratio of columns when constructing each tree and colsample_bylevel is the subsample ratio of columns for each level. Moreover, *subsample* determines the subsample training instances ratio [53].

Regularization is a process of introducing additional information to avoid overfitting, which is determined, in XGBoost, by *gamma* likewise *reg_alpha* and *reg_lambda*, applying L1 and L2 to the leaf weights. Also to control overfitting in the tree boosting process, the hyperparameter *learning_rate* and *scale_pos_weight* were tuned [53]. The hyperparameters adjusted for XGBoost in this study can be found in S1–S4 Tables.

#### CNN development and hyperparameter tuning

**Fig 1:**
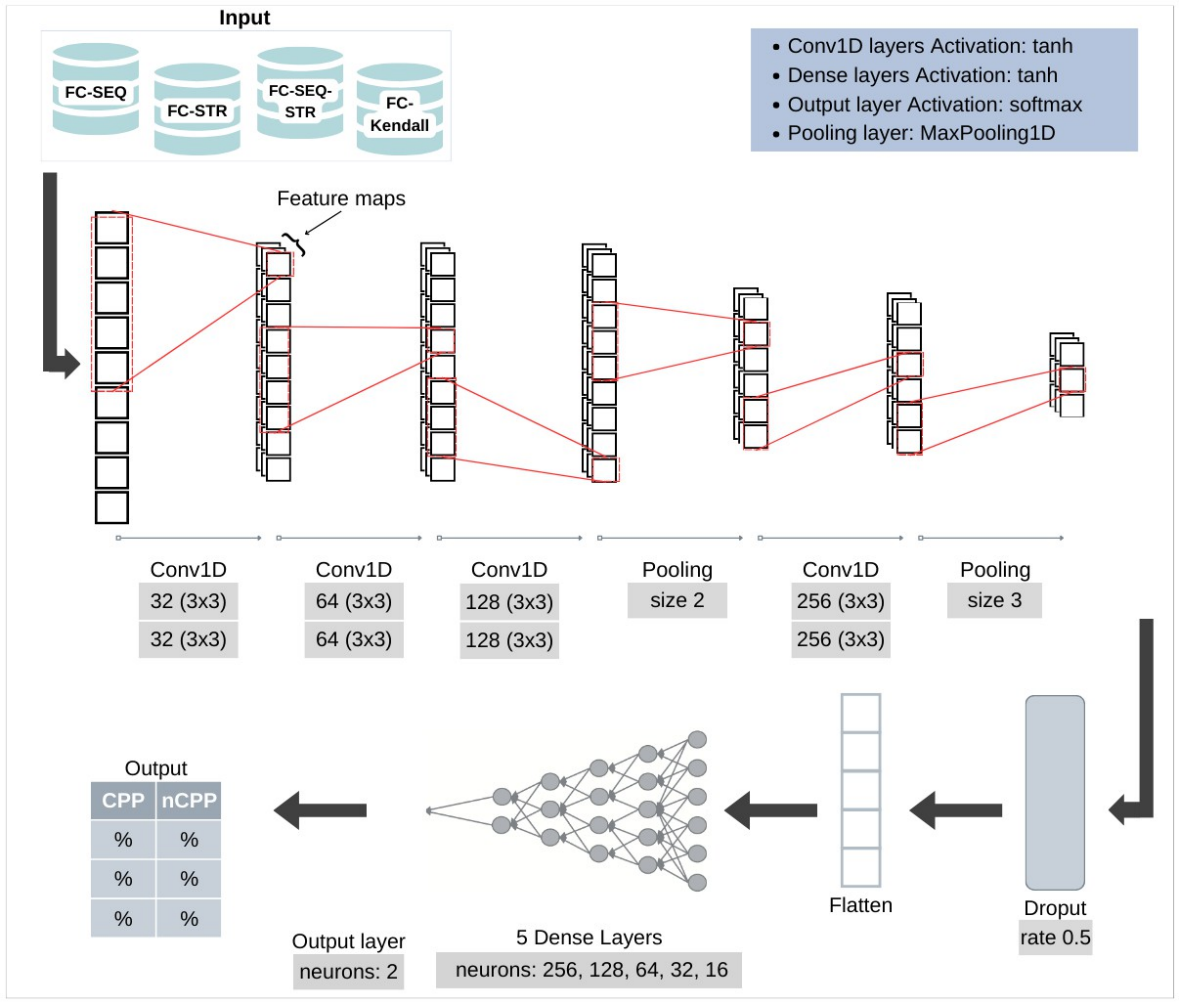
Convolutional neural network architecture developed for the ConvBoost-CPP.

The proposed CNN architecture to predict CPPs is composed of four groups of one-dimensional convolutional layers called conv1D (for tabular data) containing two layers in each group with kernel 3 × 3 and *tanh* (hyperbolic tangent) as activation function. The filter numbers in the layer groups are set to 32, 64, 128 and 256 as explained in Fig 1.

Pooling layers are added just above and just below the last group of convolutional layers to reduce the spatial dimensions of the output volume. The maximum pooling layer, used in this work, selects the maximum value in the filter range [54], where filters have sizes of 2 and 3 to respective first and last pooling layers, Fig 1. The padding *same*, used in all convolutional layers of the developed CNN, results in padding with zeros evenly left and right, above and below the data point [55].

As shown in Fig 1, the dropout layer is added before dense layers with rate of 0.5, meaning that 50% of neurons will be completely ignored during this training step, but may be active during the next one, thus preventing overfitting [54, 56]. After that, the data is flattened using the flatten layer, transforming the input matrix into an output vector.

The model described in Fig 1 also has five fully connected dense layers having 256, 128, 64, 32, and 16 neurons, respectively, using *tanh* as activation function to process their outputs. In the final CNN output layer, the *softmax* activation function is introduced, making each element of the vector belongs to a particular class of the problem, according to estimated probabilities for each class [54]. Lastly, the loss function used is *binary cross-entropy*, which penalizes models that estimate a low probability for the target class [54]. The *Adam* function is used as an optimizer for the loss function as it requires less learning rate adjustment [54].

As mentioned, the overfitting treatment in the CNN development enabled better results, and it was achieved through some hyperparameters tuning in the convolutional and dense layers, which are *activity regularizer* and *bias regularizer*, having their values exposed in Table 1. Regularization terms (regularizers) are added to the cost function that the network optimizes, allowing penalties to be applied to layer parameters or layer activity during optimization [54]. The activity regularizer is used to apply a penalty to the output and the bias regularizer applies penalties to the layer biases [55]. In the proposed CNN, activity and bias regularizer use the L1 norm to apply penalties to the output and bias in layers specified in Table 1.

**Table 1:** Activity and bias regularizers in convolutional and dense layers of the developed CNN.

Layer	Activity regularizer	Bias regularizer
Conv group 1	-	-
Conv group 2	L1(1e-3)	-
Conv group 3	L1(1e-3)	L1(1e-2)
Conv group 4	L1(1e-3)	L1(1e-2)
Dense (256)	L1(1e-3)	L1(1e-3)
Dense (128)	L1(1e-3)	L1(1e-3)
Dense (64)	L1(1e-3)	L1(1e-3)
Dense (32)	L1(1e-3)	-
Dense (16)	-	-

Regularization L1 (lasso regression) and L2 (ridge regression) owe their name to the L1 and L2 norm of a vector *W*, respectively. L1 tends to push the weights to exactly zero, leading to sparse models where all but the most important weights are zero. L2 is used for the same purpose; the difference is that the weights are reduced but never reach 0 [54]. Due to the datasets size, in the experiments identified a need to adjust the epochs to each FC to 25, 20, 25, and 20 in the respective FC-SEQ, FC-STR, FC-SEQ-STR, and FC-Kendall.

#### Construction of the ConvBoost-CPP employing the Soft Voting method

**Fig 2:**
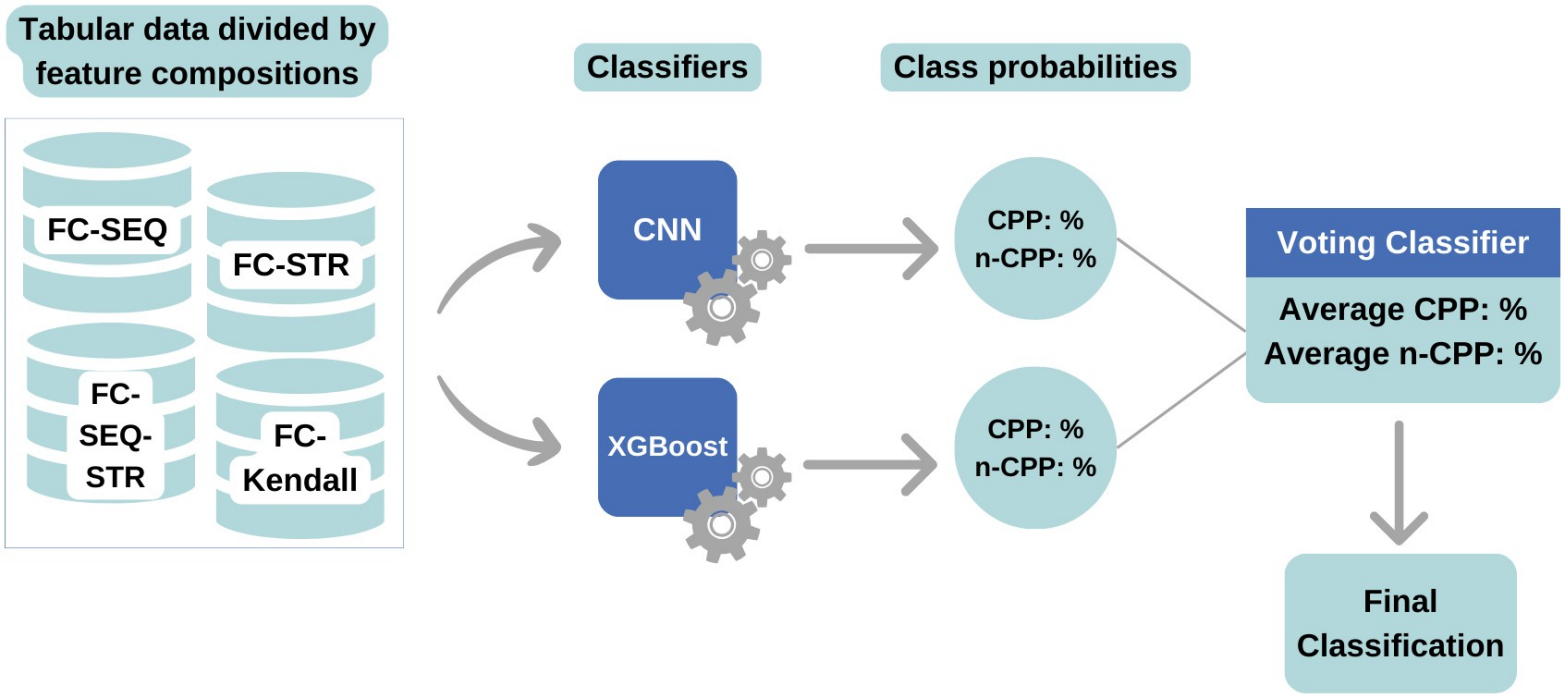
Operating flow of ConvBoost-CPP ensembling CNN and XGBoost with soft voting method.

After developing and tuning the classifiers’ hyperparameters, the Voting method was applied for classification. Fig 2 shows the workflow where XGBoost, and CNN models are subjected to the ten-fold cross-validation for constructing the voting classifier. The probabilities from each model’s ten results were used to classify the data using the soft voting technique.

The Soft Voting method, used in this research, provides a probability value that a specific peptide belongs to CPP or non-CPP (non-cell penetrating peptides). The predictions are summed and divided by the number of classifiers (average), and then the target label with the highest probability wins the vote [57].

### Model evaluation

A widely used way to evaluate ML and DL models is through a cross-validation experiment [54]. The present work employs ten-fold cross-validation to estimate the predictive performance of the proposed classifier. Through this method, the ConvBoost-CPP is compared to the classic algorithms from literature SVM, RF, GPC, Light Gradient Boosting Machine (LGBM), KNN and Multi-layer Perceptron (MLP). Then, the proposed classifier is compared to PepCNN and BChemRFCPPred, which are classifiers recently published with the purpose of classifying CPPs.

Subsequently, the FCs are compared by assessing their performance through cross-validation and independent testing. In this experiment, the cross-validation dataset, with 600 samples, is used as input to train ConvBoost-CPP and then the 150 samples for independent testing are used to test the trained model.

The evaluation metrics were chosen to verify the suitability of the model concerning the FCs, which are a) accuracy (ACC) to get the ratio of the number of correct predictions to the total number of input samples (Eq 2), b) Matthew’s correlation coefficient (MCC) to measure the difference between predicted values and actual values in a manner equivalent to the chi-square statistic in binary classifications ranging between -1 and 1 (Eq 3), c) sensitivity (SN) to evaluate the model’s ability to predict true positives (Eq 4), d) specificity (SP) to evaluate the model’s ability to predict true negatives (Eq 5), e) F1-score to verify whether the precision and recovery have approximate scores (Eq 6), and f) area under the ROC (receiver operating characteristic curve) Curve (AUC): to checking the overall model performance in classification measuring the entire two-dimensional area underneath the entire ROC curve (Eq 7), as follows:

$$ACC=\frac{TP+TN}{TP+FP+TN+FN}$$ (2)

$$MCC=\frac{TN\times TP-FN\times FP}{\sqrt{\left(TP+FP)(TP+FN)(TN+FP)(TN+FN\right)}}$$ (3)

$$SN=\frac{TP}{TP+FN}$$ (4)

$$SP=\frac{TN}{FP+TN}$$ (5)

$$F1-score=\frac{TP}{TP+\frac{FN+FP}{2}}$$ (6)

$$AUC=\int_{0}^{1}TP\;d\left(FP\right)$$ (7)

where TP is True Positive, FP is False Positive, TN is True Negative and FP is False Negative, considering the confusion matrix resulting from comparing predicted classes by the classifier and the actual values, calculated with the sklearn metrics package [51].

**Fig 3:**
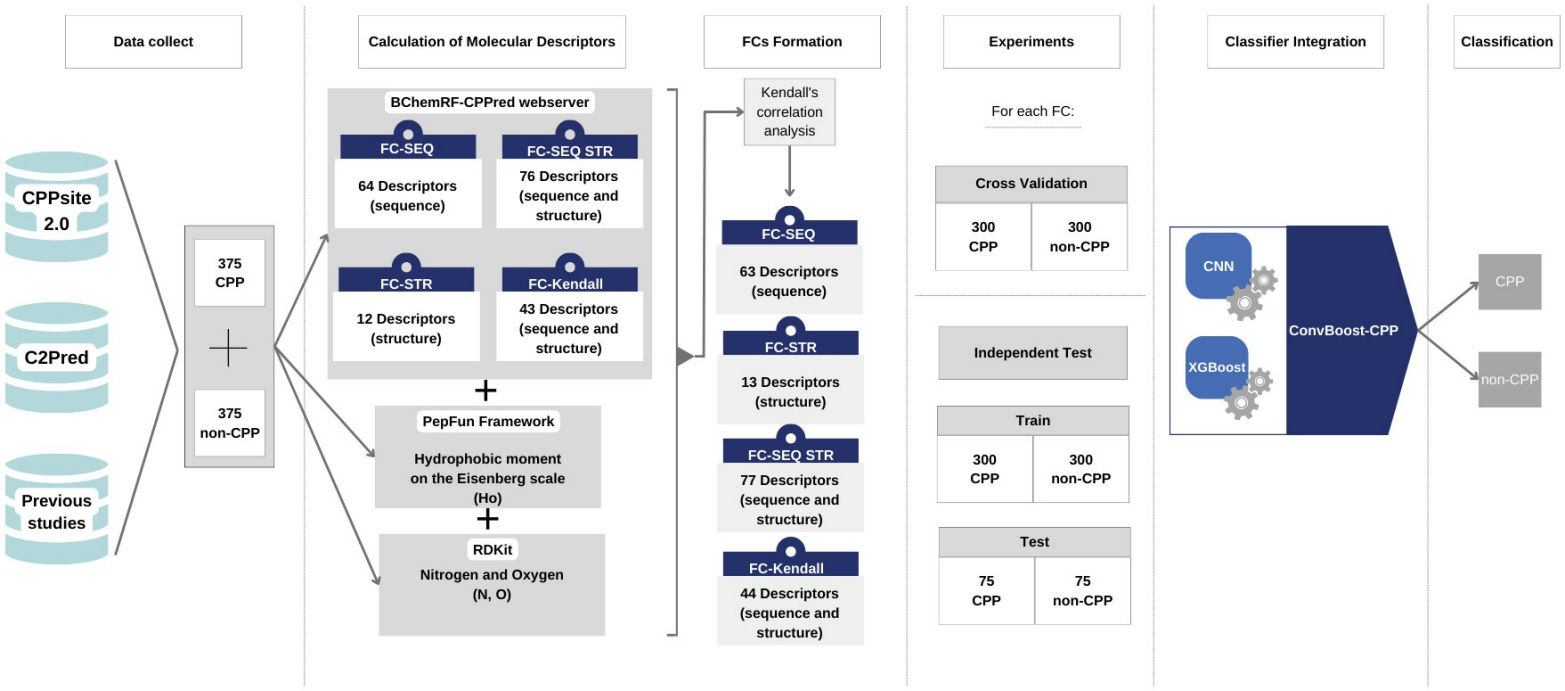
Methodological process summary used in the research development.

In summary, the methodology used in the research is represented in Fig 3 bellow. Initially, data were collected in PDB format on CPPSite2.0 [32] and C2Pred [33] servers besides previous works [11, 34, 35], which totaled 375 CPPs and 375 non-CPPs. These data were selected according to the peptides size, and afterwards the molecular descriptors were calculated with the tools BChemRF-CPPred [16], PepFun [50] and RDKit [47].

The data, then divided for training and test experiments for each FC, is used to validate the ConvBoost-CPP classifier formed by XGBoost and the improved CNN. As a result, ConvBoost-CPP classifies samples into CPP and non-CPP.

## Results and discussion

This study proposes the ConvBoost-CPP classifier, which employs the soft voting technique using XGBoost and a CNN built with overfitting treatment, taking as input sequence- and structure-based descriptors investigated to improve CPP classification. The BChem-FCs analysis (FCs calculated on the BChemRF-CPPred web server), the proposed +NOHo-FCs analysis (the proposed FCs, with N, O, Ho), and the classifiers analysis are devised through cross-validations and independent tests experiments.

### ConvBoost-CPP demonstrated a greater ability to differentiate CPPs and non-CPPs than previously published classifiers

**Fig 4:**
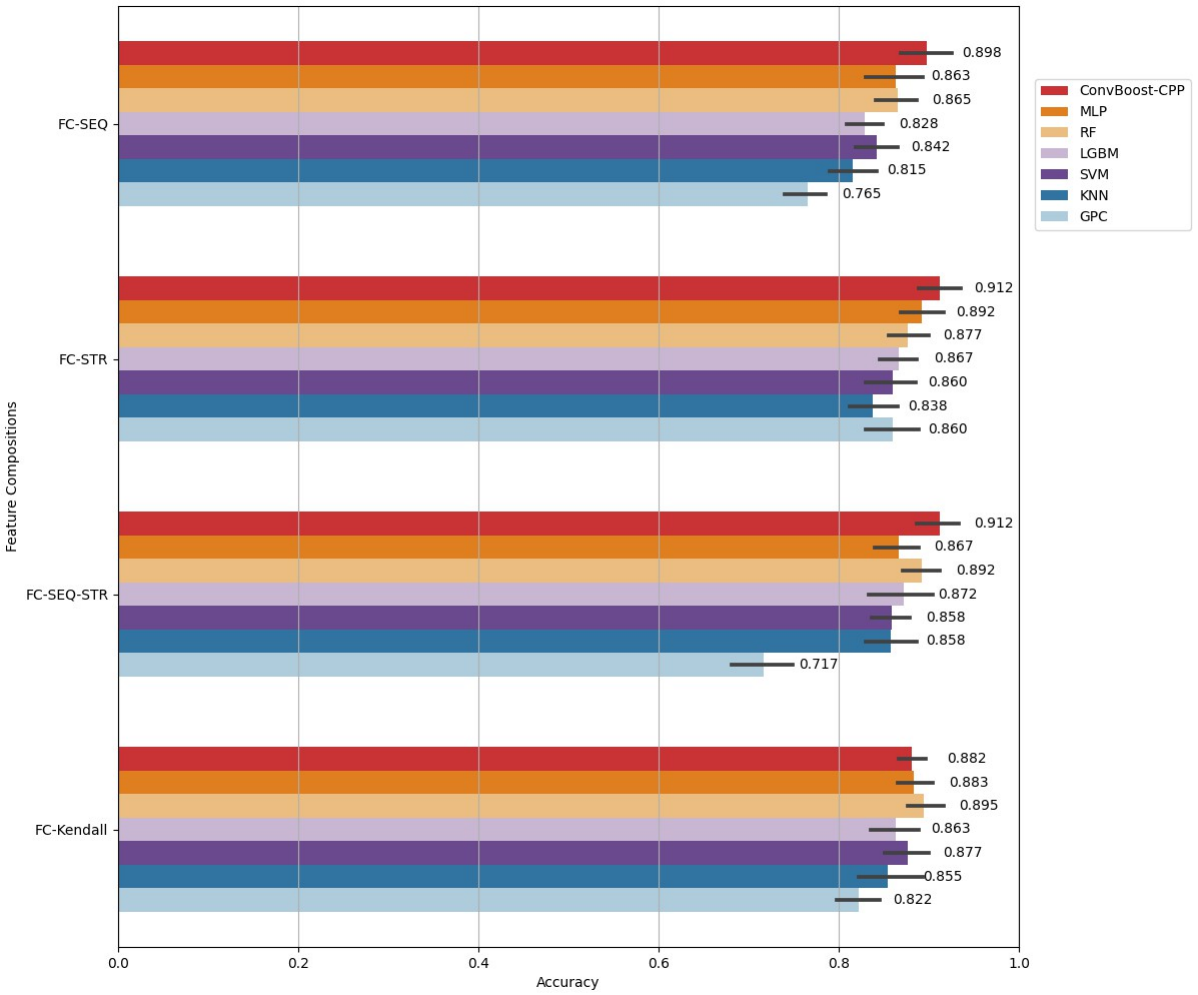
Cross-validation accuracies of ConvBoost-CPP, MLP, RF, LGBM, SVM, KNN, and GPC, using +NOHo-FCs.

Firstly, the ConvBoost-CPP was compared with the MLP, RF, LGBM, SVM, KNN and GPC algorithms. Fig 4 contrasts the average accuracies obtained for each algorithm in cross-validation using +NOHo-FCs. In this comparison, ConvBoost-CPP performed better than the other algorithms with FC-SEQ, FC-STR and FC-SEQ-STR as input.

The FC-Kendall, with 44 selected sequence- and structure-based descriptors, lead the ConvBoost-CPP, MLP, RF, and SVM to approximate accuracies. In general, GPC achieved the worst results while ConvBoost-CPP achieved the best. Moreover, the algorithms obtained the worst average accuracies with FC-SEQ as input, except GPC with the worst result obtained with FC-SEQ-STR (the largest dataset). Table 2 details the ACC, MCC, SN, SP, F1-score and AUC metrics with the respective averages achieved by the algorithms in this cross-validation experiment.

**Table 2:** Metrics scores obtained in cross-validations of ConvBoost-CPP, MLP, RF, LGBM, SVM, KNN, and GPC, using +NOHo-FCs as input.

Metric	Classifier	FC-SEQ	FC-STR	FC-SEQ-STR	FC-Kendall
ACC	ConvBoost-CPP	**0.898**	**0.912**	**0.912**	0.882
	MLP	0.863	0.832	0.867	0.883
	RF	0.865	0.877	0.892	**0.895**
	LGBM	0.828	0.867	0.872	0.863
	SVM	0.842	0.860	0.858	0.877
	KNN	0.815	0.838	0.858	0.855
	GPC	0.765	0.860	0.717	0.822
MCC	ConvBoost-CPP	**0.800**	**0.827**	**0.825**	0.767
	MLP	0.730	0.787	0.737	0.766
	RF	0.731	0.754	0.784	**0.790**
	LGBM	0.658	0.738	0.749	0.735
	SVM	0.686	0.721	0.718	0.754
	KNN	0.631	0.687	0.723	0.712
	GPC	0.547	0.729	0.485	0.647
SN	ConvBoost-CPP	**0.907**	**0.873**	0.881	0.861
	MLP	0.867	0.859	0.878	**0.883**
	RF	0.850	0.845	0.867	0.869
	LGBM	0.822	0.852	**0.895**	**0.883**
	SVM	0.823	0.821	0.838	0.868
	KNN	0.804	0.762	0.834	0.838
	GPC	0.666	0.797	0.499	0.786
SP	ConvBoost-CPP	**0.893**	**0.954**	**0.945**	0.908
	MLP	0.870	0.925	0.864	0.885
	RF	0.883	0.911	0.917	**0.923**
	LGBM	0.840	0.890	0.858	0.857
	SVM	0.866	0.896	0.883	0.887
	KNN	0.832	0.916	0.894	0.875
	GPC	0.875	0.927	0.939	0.861
F1-score	ConvBoost-CPP	**0.900**	**0.907**	**0.909**	0.878
	MLP	0.863	0.887	0.866	0.880
	RF	0.862	0.870	0.887	**0.891**
	LGBM	0.826	0.864	0.873	0.864
	SVM	0.836	0.851	0.853	0.874
	KNN	0.809	0.821	0.852	0.851
	GPC	0.734	0.850	0.635	0.814
AUC	ConvBoost-CPP	**0.900**	**0.913**	**0.913**	0.884
	MLP	0.868	0.892	0.871	0.884
	RF	0.866	0.878	0.892	**0.896**
	LGBM	0.831	0.871	0.877	0.870
	SVM	0.844	0.858	0.861	0.878
	KNN	0.818	0.839	0.864	0.857
	GPC	0.771	0.862	0.719	0.823

These scores show that ConvBoost-CPP outperforms the algorithms of literature using the FC-SEQ, FC-STR, and FC-SEQ-STR. Withal, it is notable that ConvBoost-CPP has greater ability to identify the class non-CPP, whereas SN is less than SP in almost all FCs. This effect is also observed in the results of the other algorithms, with the biggest difference between GPC’s SN (49.9%) and SP (93.9%) using FC-SEQ-STR as input, which implies its low F1-score.

Table 2 also reveals that RF achieves the best scores among the algorithms with approximate accuracies using FC-Kendall. However, considering the other FCs, its performance is similar to other ML algorithms. In contrast, ConvBoost-CPP presents greater performances with the other set of descriptors, whether based only on sequence or structure, or a set of them; and either with the biggest (FC-SEQ-STR, 76 descriptors), or with the smallest (FC-STR, 13 descriptors) FC.

These results show that the proposed classifier has an improved ability to correctly differentiate non-CPPs from CPPs. Furthermore, analyzing the scores obtained with the FC-STR, this FC has the highest MCC (0.827), one of the greater accuracies (91.2%) and F1-score (90.7%), proving that ConvBoost-CPP is the best classifier among the seven analyzed, employing the smallest set of descriptors to classify CPPs.

**Fig 5:**
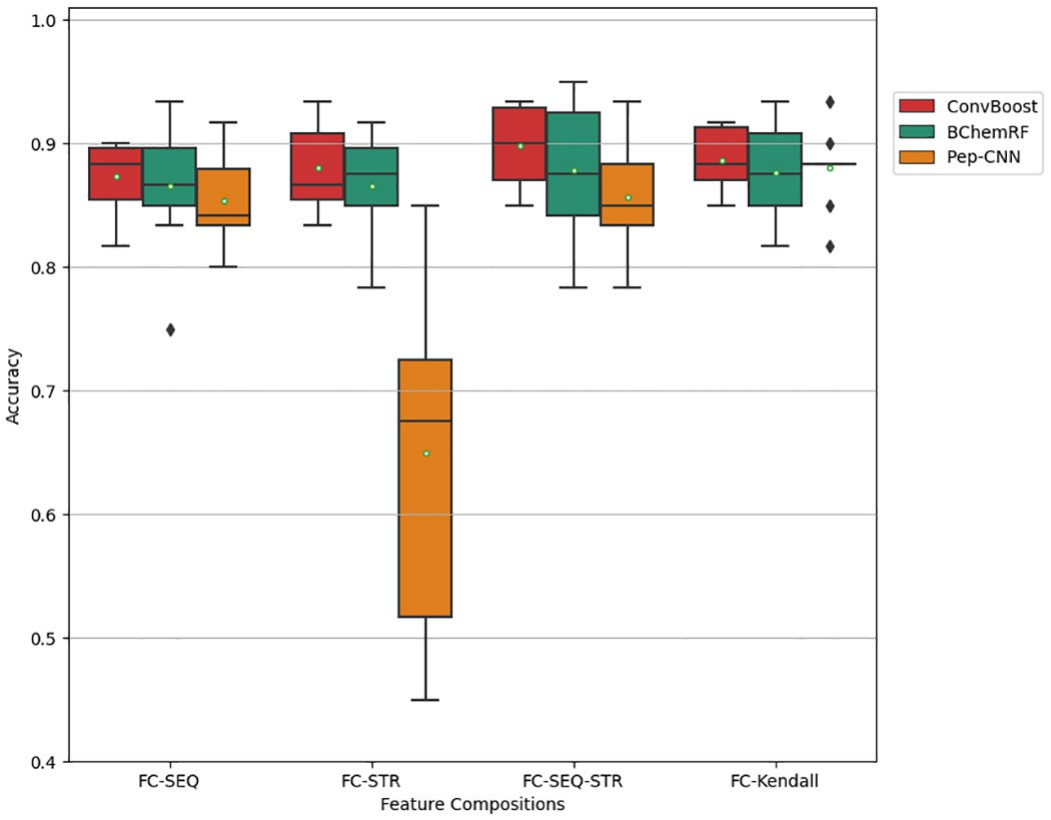
Cross-validation accuracies of ConvBoost-CPP (red), BChemRF (green), and Pep-CNN (orange) using BChem-FCs.

Thereafter, ConvBoost-CPP was compared with BChemRF classifier [16], using the results available in the published work, and Pep-CNN [9], reproducing that proposed CNN for peptide classification. In this experiment, which is analyzed in Fig 5, the BChem-FCs were used as input to fairly compare the algorithms, since the results collected were achieved with these sets. The plotted boxplot graphs for the further analyses have their standard structure with the inclusion of the accuracy average, represented by white dots on the respective boxplots to provide better visualization of cross-validation results.

Fig 5 shows that ConvBoost-CPP reaches superior accuracies than the previously published frameworks, with greater advantage when FC-SEQ-STR, with 76 sequence- and structure-based descriptors, is used as input. On the other hand, Pep-CNN and BChemRF achieve better results with FC-Kendall as input, containing 43 descriptors resulting from an optimized selection of structure- and sequence-based descriptors according to Kendall’s correlation analysis.

Fig 5 also shows that the worst structure is Pep-CNN using FC-STR from BChem-FCs as input. Table 3 explains the average accuracy achieved by these classifiers in cross-validations using BChem-FCs as input.

**Table 3:** Average accuracies achieved by ConvBoost-CPP, BChemRF, and Pep-CNN using BChem-FCs in cross-validation experiments.

Classifiers	FC-SEQ	FC-STR	FC-SEQ-STR	FC-Kendall
ConvBoost-CPP	**0.873**	**0.880**	**0.898**	**0.886**
BChemRF	0.865	0.865	0.873	0.876
Pep-CNN	0.853	0.650	0.856	0.880

Although the classifiers have similar average accuracy using FC-Kendall, ConvBoost-CPP overall performs better using all BChem-FCs than the other algorithms, besides presenting greater classification consistency in the ten-fold cross-validation in Fig 5.

### The descriptors N, O, and Ho provided improvements in the classification of CPPs

To investigate the influence of descriptors N, O, and Ho on CPPs classification, initially, four FCs were generated in BChemRF-CPPred web server: FC-SEQ (64 descriptors, only sequence-based), FC-STR (12 descriptors, only structure-based), FC-SEQ-STR (76 sequence-based and structure-based descriptors), and FC-Kendall (a selection of 43 structure- and sequence-based descriptors according to Kendall’s correlation analysis). The three descriptors were added to FC-STR, FC-SEQ-STR, and FC-Kendall, and then all FCs underwent a Kendall’s correlation analysis to remove redundant features.

**Fig 6:**
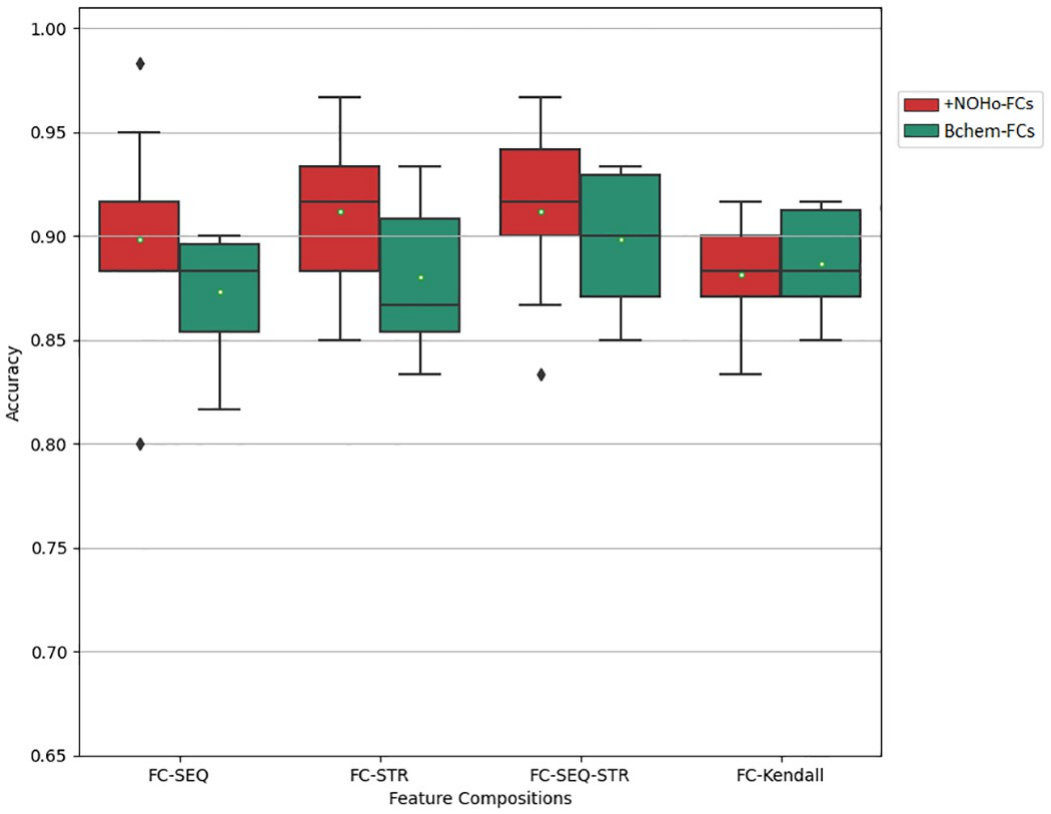
Cross-validation accuracies of ConvBoost-CPP using +NOHo-FCs (red) and ConvBoost-CPP using BChem-FCs (green).

The prediction performance of ConvBoost-CPP in ten-fold cross-validation using the +NOHo-FCs and BChem-FCs is observed in Fig 6. It is evident that, with the sole exception of FC-Kendall, there is a significant improvement in the ability of the developed model to correctly differentiate non-CPPs and CPPs using +NOHo-FCs, mainly FC-STR containing ten structure-based descriptors with the addition of the descriptors N, O, and Ho.

Table 4 consists of metrics calculated from the results obtained by ConvBoost-CPP in cross-validations using the FCs generated by the BChemRF web server and the proposed ones. It can be seen that, among the BChem-FCs, ConvBoost-CPP performs better with FCs containing sequence- and structure-based descriptors, with emphasis on the FC-SEQ-STR. Nevertheless, among the proposed FCs, ConvBoost-CPP achieves better results with FC-SEQ-STR and FC-STR as input, with approximate scores across all metrics.

**Table 4:** Metrics scores obtained in cross-validations of ConvBoost-CPP using BChem-FCs and +NOHo-FCs as input.

Metric	Set of FCs	FC-SEQ	FC-STR	FC-SEQ-STR	FC-Kendall
ACC	BChem-FCs	0.873	0.880	0.898	**0.886**
	+NOHo-FCs	**0.898**	**0.912**	**0.912**	0.882
MCC	BChem-FCs	0.750	0.759	0.790	**0.770**
	+NOHo-FCs	**0.800**	**0.827**	**0.825**	0.767
SN	BChem-FCs	0.854	0.851	0.873	**0.864**
	+NOHo-FCs	**0.907**	**0.873**	**0.881**	0.861
SP	BChem-FCs	**0.899**	0.910	0.924	**0.910**
	+NOHo-FCs	0.893	**0.954**	**0.945**	0.908
F1-score	BChem-FCs	0.869	0.872	0.893	**0.882**
	+NOHo-FCs	**0.900**	**0.907**	**0.909**	0.878
AUC	BChem-FCs	0.877	0.880	0.898	0.883
	+NOHo-FCs	**0.900**	**0.913**	**0.913**	**0.884**

Considering the two groups of FCs, we see that the new FC-SEQ and FC-STR had an enhancement between 3% and 5% over their respective BChem-FCs, while FC-SEQ-STR and FC-Kendall had an enhancement between 1% and 3%. Also, comparing the accuracy reached by ConvBoost-CPP using the +NOHo-FCs and the accuracy achieved by BChemRF-CPPred using the BChem-FCs, there is a visible enhancement mainly in the FC-STR.

An important point to be highlighted in this analysis is the number of descriptors necessary to effect the classification with better accuracy. While the other FCs contain between 44 and 77 descriptors, the new FC-STR is less complex having only 13 features. Furthermore, the difference between BChem-FCs and +NOHo-FCs regarding the descriptors quantity is of just one feature, since three descriptors were added to FC-STR, FC-SEQ-STR, and FC-Kendall, two descriptors were removed from them with Kendall’s correlation analysis, and from FC-SEQ only one descriptor was removed.

Focusing on metrics of FC-STR in Table 4, this FC had the greatest improvement in MCC, F1-score, and AUC, indicating that overall, the classifier performs the differentiation of CPPs and non-CPPs coherently, considering true positives and true negatives, using only structure descriptors with N, O, and Ho. Moreover, observing the SP metric of the FC-STR from +NOHo-FCs, it is possible to state that this is the best FC to identify the class CPP, for having achieved the best specificity average (95.4%).

Regarding the accuracies obtained in independent tests shown in Table 5, it is notable that the descriptors N, O, and Ho, besides Kendall’s correlation analysis, cause a positive impact on the classification of CPPs aggregated to some previously published descriptors in the BChem web server, providing better data patterns for the classifier to recognize CPPs and non-CPPs. The +NOHo-FCs reach better accuracies in all FCs, especially FC-STR and FC-SEQ-STR, which also performed better in cross-validation.

**Table 5:** Metrics scores obtained in independent tests of ConvBoost-CPP using BChem-FCs and +NOHo-FCs as input.

Metric	Set of FCs	FC-SEQ	FC-STR	FC-SEQ-STR	FC-Kendall
ACC	BChem-FCs	0.873	0.826	0.866	0.886
	+NOHo-FCs	**0.880**	**0.913**	**0.920**	**0.893**
MCC	BChem-FCs	0.747	0.657	0.734	0.775
	+NOHo-FCs	**0.762**	**0.828**	**0.841**	**0.789**
SN	BChem-FCs	0.867	0.773	0.840	**0.853**
	+NOHo-FCs	**0.840**	**0.943**	**0.944**	**0.853**
SP	BChem-FCs	0.880	0.880	0.893	0.920
	+NOHo-FCs	**0.920**	**0.947**	**0.947**	**0.933**
F1-score	BChem-FCs	0.872	0.817	0.863	0.882
	+NOHo-FCs	**0.875**	**0.910**	**0.918**	**0.889**
AUC	BChem-FCs	0.873	0.826	0.866	**0.886**
	+NOHo-FCs	**0.880**	**0.913**	**0.920**	0.893

The FC-Kendall performs best among the Bchem-FCs, with 43 descriptors resulting from an optimized selection of sequence- and structure-based descriptors. However, unlike FC-SEQ and FC-Kendall, FC-STR and FC-SEQ-STR present, in this experiment, information that allows the classifier to identify the CPPs and non-CPPs classes at high rates, around 94%, considering SN and SP metrics.

Although the new FC-SEQ-STR had reached an average accuracy slightly better than the new FC-STR, the Kruskal–Wallis test (*p value* = 0.92048) showed no statistically significant difference between this metric obtained by ConvBoost-CPP using these FCs. The F1-score, MCC, and AUC metrics, obtained by these two FCs among the +NOHo-FCs, demonstrate that we can state that these two sets provide information of similar importance in the classification of CPPs. Thus, FC-STR got the best performance considering its dimension with only 13 descriptors, besides the fact that it only contains structure descriptors.

We also compared the structure for CPP classification developed in this work with the structure developed for BChemRF-CPPred in Table 6. Considering the BChemRF results obtained in independent tests using BChem-FCs as input and ConvBoost-CPP using +NOHo-FCs as input, the new structure outperforms BChemRF results.

**Table 6:** Independent test accuracies achieved by BchemRF using BChem-FCs and ConvBoost-CPP using +NOHo-FCs as input.

Structure	FC-SEQ	FC-STR	FC-SEQ-STR	FC-Kendall
BChemRF with BChem-FCs	0.853	0.886	0.873	**0.906**
ConvBoost-CPP with +NOHo-FCs	**0.880**	**0.913**	**0.920**	0.893

Despite the FC-Kendall from BChem-FCs being an optimized selection of 43 sequence- and structure-based descriptors, this set does not promote superior results compared to the use of just 13 structure-based descriptors. Thus, the FC-STR features are identified as the best molecular descriptors used to differentiate CPPs and non-CPPs.

In general, we can see in Table 6 that the structure developed here surpasses the previously published structure not only in terms of accuracy but also by demonstrating that the smallest FC (FC-STR with 13 descriptors) provides as valuable information for classifying CPPs as the largest FC (FC- SEQ-STR with 77 descriptors), even FC-STR having only structure-based descriptors, unlike FC-SEQ-STR composed of all sequence and structure descriptors investigated.

## Conclusion

It was demonstrated in this work that with the addition of the structure descriptors N, O, and Ho in the FCs encompassing this class of descriptors there is an improvement in the CPPs classification performance. Furthermore, the overfitting treatment applied to CNN contributed to the classifications made by ConvBoost-CPP becoming more accurate, as in some instances, it was observed that CNN boosted the correct classification result.

The proposed ConvBoost-CPP cooperatively with the structure-based FC-STR containing the descriptors tPSA, cLogP, HBA, NAR, NRB, *Fsp*^3^, NPA, NG, NetC, and NNCAA with the addition of N, O, and Ho descriptors, produces superior results in classifying CPPs compared to other FCs and previously published frameworks, with the accuracy achieved by this structure of 91.3% in cross-validation and independent testing. These results not only proved that our tool has a greater ability to correctly predict CPPs, as the descriptors encompassed in the FC-STR produced in this research provide more significant information for the CPP prediction.

Although FC-SEQ-STR achieves results statistically similar to those of FC-STR, the number of descriptors used in this approach is considerably higher, requiring more computational resources for prediction and being more complex for experts to investigate the descriptors singularly in this research line. Additionally, FC-STR demonstrated, in cross-validations, to be better at identifying CPPs, achieving the highest average in the SP metric.

In conclusion, the CPPs classification can be performed using a few descriptors, assuming structure descriptors and chemical elements, such as N, O and Ho, that can significantly impact the permeation of these molecules in the lipid bilayer membrane. Furthermore, the investigation of descriptors used in classification analyzes of other classes of peptides can further improve the task of classifying CPPs, considering the similarities of these molecules.

## Supporting information

S1 Table Hyperparameters of the best model of XGBoost using FC-SEQ as input. Hyperparameter: hyperparameter’s name. Value: value used in the hyperparameter.
(PDF)

S2 Table Hyperparameters of the best model of XGBoost using FC-STR as input. Hyperparameter: hyperparameter’s name. Value: value used in the hyperparameter.
(PDF)

S3 Table Hyperparameters of the best model of XGBoost using FC-SEQ-STR as input. Hyperparameter: hyperparameter’s name. Value: value used in the hyperparameter.
(PDF)

S4 Table Hyperparameters of the best model of XGBoost using FC-Kendall as input. Hyperparameter: hyperparameter’s name. Value: value used in the hyperparameter.
(PDF)

S5 Table Molecular descriptors used in feature compositions. Molecular descriptor: Molecular descriptor nomenclature. Description: Meaning of the nomenclature or description of the molecular descriptor. Based on: which class the descriptor belongs to among those studied.
(PDF)
