# Supplementary material for: Investigating molecular descriptors in cell-penetrating peptides prediction with deep learning: Employing N, O, and hydrophobicity according to the Eisenberg scale
Source: PLoS One. 2024 Jun 13;19(6):e0305253. doi: 10.1371/journal.pone.0305253 (PMC11175476; doi:10.1371/journal.pone.0305253)
Supplement: S1 Table — Hyperparameter: hyperparameter’s name. Value: value used in the hyperparameter. (PDF) [file pone.0305253.s001.pdf]

Table S1. Hyperparameters of the best model of XGBoost using FC-SEQ as input. **Hyperparameter:** hyperparameter's name. **Value:** value used in the hyperparameter.

Hyperparameter	Value
colsample_bylevel	0.8506899781724641
colsample_bytree	0.17219010848522695
gamma	1.675823794985098e-05
learning_rate	0.028014182714153124
max_delta_step	0
max_depth	16
min_child_weight	0.7059430081342065
n_estimators	194
reg_alpha	1.2905368590789031e-07
reg_lambda	0.009433462784709972
scale_pos_weight	0.6177491952898517
subsample	0.857344708916481
tree_method	'approx'
