# Supplementary material for: Investigating molecular descriptors in cell-penetrating peptides prediction with deep learning: Employing N, O, and hydrophobicity according to the Eisenberg scale
Source: PLoS One. 2024 Jun 13;19(6):e0305253. doi: 10.1371/journal.pone.0305253 (PMC11175476; doi:10.1371/journal.pone.0305253)
Supplement: S3 Table — Hyperparameter: hyperparameter’s name. Value: value used in the hyperparameter. (PDF) [file pone.0305253.s003.pdf]

Table S3. Hyperparameters of the best model of XGBoost using FC-SEQ-STR as input. **Hyperparameter**: hyperparameter's name. **Value**: value used in the hyperparameter.

Hyperparameter	Value
colsample_bylevel	0.8571776924171046
colsample_bytree	0.9617455291900485
gamma	0.038054991622747364
learning_rate	0.06261617692921269
max_delta_step	1
max_depth	4
min_child_weight	0.181016026397066
n_estimators	380
reg_alpha	5.951660631505972e-07
reg_lambda	3.300012890326377e-08
scale_pos_weight	0.17895820140127336
subsample	0.9934995154106276
tree_method	'auto'
