# Supplementary material for: Investigating molecular descriptors in cell-penetrating peptides prediction with deep learning: Employing N, O, and hydrophobicity according to the Eisenberg scale
Source: PLoS One. 2024 Jun 13;19(6):e0305253. doi: 10.1371/journal.pone.0305253 (PMC11175476; doi:10.1371/journal.pone.0305253)
Supplement: S4 Table — Hyperparameter: hyperparameter’s name. Value: value used in the hyperparameter. (PDF) [file pone.0305253.s004.pdf]

Table S4. Hyperparameters of the best model of XGBoost using FC-Kendall as input. **Hyperparameter**: hyperparameter's name. **Value**: value used in the hyperparameter.

Hyperparameter	Value
colsample_bylevel	0.35
colsample_bytree	0.8504230806447873
gamma	6.964110402415467e-05
learning_rate	0.08440117919133446
max_delta_step	19
max_depth	16
min_child_weight	1.4213089219872137
n_estimators	185
reg_alpha	1.366354168505398e-06
reg_lambda	0.0004634492281691692
scale_pos_weight	0.7065131611930926
subsample	0.8774566112271143
tree_method	'hist'
