# Supplementary material for: Investigating molecular descriptors in cell-penetrating peptides prediction with deep learning: Employing N, O, and hydrophobicity according to the Eisenberg scale
Source: PLoS One. 2024 Jun 13;19(6):e0305253. doi: 10.1371/journal.pone.0305253 (PMC11175476; doi:10.1371/journal.pone.0305253)
Supplement: S5 Table — Molecular descriptor: Molecular descriptor nomenclature. Description: Meaning of the nomenclature or description of the molecular descriptor. Based on: which class the descriptor belongs to among those studied. (PDF) [file pone.0305253.s005.pdf]

**Table S5**. Molecular descriptors used in feature compositions. **Molecular descriptor**: Molecular descriptor nomenclature. **Description**: Meaning of the nomenclature or description of the molecular descriptor. **Based on**: which class the descriptor belongs to among those studied.

| Molecular descriptor | Description | Based on |
|---|---|---|
| N | Nitrogen | Structure |
| O | Oxygen | Structure |
| Ho | Hydrophobicity according to the Eisenberg scale | Structure |
| MW | Molecular weigh | Structure |
| tPSA | Topological polar surface are | Structure |
| cLogP | 1-octanol/water partition coefficient | Structure |
| HBA | Hydrogen bond acceptor | Structure |
| HBD | Hydrogen bond donor | Structure |
| NAR | Number of aromatic rings | Structure |
| NRB | Number of rotatable bond | Structure |
| Fsp3 | Fraction of sp3-hybridized carbon atoms | Structure |
| NPA | Number of primary amino groups | Structure |
| NG | Number of guanidinium groups | Structure |
| NetC | Net charge | Structure |
| NNCAA | Number of negatively charged amino acids | Structure |
| f[ARG] | Fraction of Arginine | Sequence |
| f[LYS] | Fraction of Lysine | Sequence |
| PAAC1 | Pseudo-Amino Acid 1 | Sequence |
| PAAC2 | Pseudo-Amino Acid 2 | Sequence |
| PAAC3 | Pseudo-Amino Acid 3 | Sequence |
| PAAC4 | Pseudo-Amino Acid 4 | Sequence |
| PAAC5 | Pseudo-Amino Acid 5 | Sequence |
| PAAC6 | Pseudo-Amino Acid 6 | Sequence |
| PAAC7 | Pseudo-Amino Acid 7 | Sequence |
| PAAC8 | Pseudo-Amino Acid 8 | Sequence |
| PAAC9 | Pseudo-Amino Acid 9 | Sequence |
| PAAC10 | Pseudo-Amino Acid 10 | Sequence |
| PAAC11 | Pseudo-Amino Acid 11 | Sequence |
| PAAC12 | Pseudo-Amino Acid 12 | Sequence |
| PAAC13 | Pseudo-Amino Acid 13 | Sequence |
| PAAC14 | Pseudo-Amino Acid 14 | Sequence |
| PAAC15 | Pseudo-Amino Acid 15 | Sequence |

| | | |
|---|---|---|
| PAAC16 | Pseudo-Amino Acid 16 | Sequence |
| PAAC17 | Pseudo-Amino Acid 17 | Sequence |
| PAAC18 | Pseudo-Amino Acid 18 | Sequence |
| PAAC19 | Pseudo-Amino Acid 19 | Sequence |
| PAAC20 | Pseudo-Amino Acid 20 | Sequence |
| PAAC21 | Pseudo-Amino Acid 21 | Sequence |
| PAAC22 | Pseudo-Amino Acid 22 | Sequence |
| GL | Glycine-Leucine | Sequence |
| GF | Glycine-Phenylalanine | Sequence |
| LG | Leucine-Glycine | Sequence |
| GA | Glycine-Alanine | Sequence |
| VC | Valine-Cysteine | Sequence |
| CC | Cysteine-Cysteine | Sequence |
| FL | Phenylalanine-Leucine | Sequence |
| GT | Glycine-Threonine | Sequence |
| CV | Cysteine-Valine | Sequence |
| SE | Serine-Glutamic Acid | Sequence |
| LS | Leucine-Serine | Sequence |
| GP | Glycine-Proline | Sequence |
| FG | Phenylalanine-Glycine | Sequence |
| FC | Phenylalanine-Cysteine | Sequence |
| TV | Threonine-Valine | Sequence |
| GI | Glycine-Isoleucine | Sequence |
| PL | Proline-Leucine | Sequence |
| PG | Proline-Glycine | Sequence |
| CN | Cysteine-Asparagine | Sequence |
| ET | Glutamic Acid-Threonine | Sequence |
| QN | Glutamine-Asparagine | Sequence |
| RL | Arginine-Leucine | Sequence |
| IW | Isoleucine-Tryptophan | Sequence |
| RM | Arginine-Methionine | Sequence |
| GR | Glycine-Arginine | Sequence |
| QR | Glutamine-Arginine | Sequence |
| RI | Arginine-Isoleucine | Sequence |
| RW | Arginine-Tryptophan | Sequence |
| FQ | Phenylalanine-Glutamine | Sequence |

| | | |
|---|---|---|
| RS | Arginine-Serine | Sequence |
| WF | Tryptophan-Phenylalanine | Sequence |
| KW | Lysine-Tryptophan | Sequence |
| NR | Asparagine-Arginine | Sequence |
| WK | Tryptophan-Lysine | Sequence |
| WR | Tryptophan-Arginine | Sequence |
| RK | Arginine-Lysine | Sequence |
| RQ | Arginine-Glutamine | Sequence |
| KR | Lysine-Arginine | Sequence |
| KK | Lysine-Lysine | Sequence |
| RR | Arginine-Arginine | Sequence |
